# The MapMe Body Scales: Validity and Reliability of a Biometrically Accurate, Photorealistic Set of Child Body Size Scales

**DOI:** 10.3390/children11101243

**Published:** 2024-10-16

**Authors:** Bethany J. Ridley, Elizabeth H. Evans, Piers L. Cornelissen, Robin S. S. Kramer, Martin J. Tovée

**Affiliations:** 1 Department of Psychology, Northumbria University, Newcastle upon Tyne NE1 8ST, UK; bethany.j.ridley@northumbria.ac.uk (B.J.R.); piers.cornelissen@northumbria.ac.uk (P.L.C.); 2 Department of Psychology, Durham University, Durham DH1 3LE, UK; elizabeth.evans@durham.ac.uk; 3 School of Psychology, University of Lincoln, Lincoln LN6 7TS, UK; rkramer@lincoln.ac.uk

**Keywords:** childhood weight, overweight, body size perception, BMI categories

## Abstract

Background/Objectives: It is vital to identify children whose weight status means that they may benefit from medical or behavioural support, but adult visual judgements of child weight status are inaccurate, and children are seldom routinely weighed and measured. Consequently, there is a need for validated visual tools for use in training, communication, and interventions relating to child weight. Methods: This paper presents validation data for a set of innovative photo-realistic colour body size scales depicting boys and girls aged 4–5 and 10–11. Each age- and gender-specific scale consists of 7 figures based on three-dimensional (3D) scans of 388 children to accurately represent the change in body size caused by changing adiposity. To assess scale validity, 238 adult participants (105 men, 132 women, 1 non-binary individual) undertook two tasks: rating figure adiposity using a visual analogue scale and ranking figures in ascending order of adiposity (OSF Reference: gdp9j). Results: Participants accurately estimated the relative adiposity of each figure, i.e., they were able to tell the difference between figures and correctly rank them by size. This demonstrates scale validity for use in body size tasks. One hundred and fifty-one participants also provided 3-day test–retest data, which demonstrates excellent short-term reliability. Conclusions: Overall, the MapMe child body size scales provide an anthropometrically accurate, valid, reliable, and usable tool for size-related tasks and communication with adults regarding child weight.

## 1. Introduction

Both overweight and obesity (OWO) and underweight in children are serious global public health challenges, affecting an increasing proportion of children [1,2,3]. OWO predicts potentially serious health consequences over the lifespan [4], such as selected cancers, cardiovascular disease, type 2 diabetes, and mental health difficulties [5,6]. Children with OWO are also more likely to experience social isolation, poor self-image, and poor academic performance [7]. Whilst less common in high-income countries, underweight in children nevertheless predicts childhood health risks, including increased vulnerability to infections, poorer general health, nutritional deficiencies, decreased cognitive performance, and reduced psychological well-being (e.g., [8,9,10]). Detection of both OWO and underweight in children is, therefore, a very high public health priority.

### 1.1. An Inability to Detect Unhealthy Weight in Children

Family and school-based interventions can support families in reducing childhood OWO [11,12] or help children in the underweight range gain weight [13], but this requires recognition that weight-related support is needed (e.g., [14]). In many countries, children are not routinely weighed and measured. Even when they are, weight problems can develop between measurement points, and parents are often sceptical of the accuracy of BMI data for children. Moreover, visual detection of both OWO and underweight in children is generally poor amongst parents [15,16,17], healthcare professionals (HCPs; [18,19]) and members of the general population without children [20]. Specifically, observers tend to categorise a child’s weight as mid-range (‘healthy’ or ‘recommended’ weight), even when objective measurements based on age- and sex-standardised growth reference values indicate it is higher or lower. There is, therefore, a need for resources to support both detection (e.g., recognition that medical weight support may be needed) and communication about higher and lower child weight.

### 1.2. How Can Visual Tools Help?

Visual tools which accurately depict children of different weight statuses may help reduce under-detection of high or low child weight status by enabling parents and HCPs to calibrate their weight judgements against standardised, objective stimuli [21]. This is a promising approach because parents and often HCPs initially rely upon visual judgements before deciding (if at all) to weigh and measure a child [18]. Moreover, both parents and HCPs report an interest in image-facilitated conversations about child weight [22,23,24]. Existing detection-focused communication tools [25] tend to be based on BMI centile charts. Whilst anthropometric measurement remains the gold standard for detecting high/low weight, such charts are seldom used in paediatric medical contexts [26]. HCPs report that they often lack appropriate measuring equipment [27] and express concerns about low parental understanding of growth charts [28]; indeed, parents do frequently struggle to interpret such charts [28,29]. Simplified versions of BMI charts (e.g., ‘My Weight Ruler’, [30]) have been developed to address this, and these show a moderate degree of clinical utility but still require HCP involvement [25]. In contrast, visual tools based on pictures of children may be easier for parents to intuitively interpret, independent from HCP input, facilitating weight status detection. Image-based resources are also helpful in communicating information rapidly but neutrally, especially when there are language barriers [31]. In addition, people—notably parents, but also some HCPs—report a preference for visual cues and comparisons to detect child weight status [32,33] rather than objective measurements of weight and height (e.g., [21,32]). Overall, therefore, image-based tools have the potential to be effectively used in educational interventions and parental communication, aiming to improve the detection of high/low weight and support communication with children and families [31].

Visual representations of various child weight statuses have previously been developed for research purposes [34,35,36]. Whilst these instruments represent important steps forward in exploring the role of visual tools for weight-related detection and communication, they also show significant limitations. None of them are directly based on representative biometric data for a particular population. Instead, they are usually based on an artist’s interpretation of a particular weight category. For example, Reifsnider et al.’s (2006) child silhouettes were unrealistic because they resembled adult body proportions [36]. This is inappropriate given that adult body proportions and shape are relatively stable, but children’s bodies are constantly changing [37]. So, it is important to use age-appropriate body scales [38] and not simply use adult scales in the judgement of child weight [39]. Both Hager et al.’s (2010) toddler silhouettes and Eckstein et al.’s (2006) sketches were produced by artists [34,35]. In neither case did each image correspond to a specific BMI point, and nor was a full range of body size represented. This lack of realism, combined with the absence of anthropometric accuracy, undermines their utility and acceptability to parents [21]. The use of line drawings and silhouettes fails to reproduce a range of visual cues to stomach depth and trunk volume, which are important signals of adiposity and body [40,41]. Huang et al. (2007) [42] and Truby and Paxton (2002) [43] used photographs of children of known body weight, but in neither case does the resolution or angle of images allow optimal visual detection of subtle adiposity cues. Another key critique of previous visual tools is that they assume that images showing White child body size and shape variation can be applied across different ethnicities despite evidence of anthropometric variability in child body composition and adiposity distribution [44,45]. There is, therefore, a need to ensure that images match the ethnicity of the children in question, as highlighted by parent feedback in Jones et al., 2018 [21].

To address these limitations, we previously used computer-generated imaging (CGI) techniques to create photorealistic colour figural scales based on 388 three-dimensional (3D) scans of 4–5 and 10–11-year-old children to represent the change in body size caused by changing adiposity (The MapMe Child Body Scales; [21]) Each age- and gender-matched scale consisted of seven figures. We used these scales in a pilot randomised controlled trial of educational materials to effectively improve weight outcomes for children with OWO [21]. Although focus group testing showed the scales were acceptable to parents, they were not assessed for reliability or validity in testing the perception of a child’s body weight. In the current study, therefore, we evaluated both the reliability and validity of an updated version of these stimuli. Notably, using the same database of 3D scans, we applied new analytic techniques (for technical details, see [46]) and image rendering to produce clearer, more accurate and more realistic CGI images, now presented at a three-quarter viewing angle. Evidence shows that both adult and child body weight judgements are more accurate for figures in this orientation (e.g., [20,41]).

### 1.3. Study Rationale

To evaluate the validity and reliability of the revised MapMe-2 child body size scales, participants in the current study completed two behavioural tasks. For the figures to show validity, participants needed to be able to (a) distinguish between one figure and another and (b) accurately *directionally* compare figures, i.e., see that one was larger/smaller than the next. In the first task, participants rated the adiposity of figures using a visual analogue scale (VAS), which ranged from ‘very underweight’ to ‘very overweight’. In the second task, they ranked the bodies within each scale in ascending order of perceived adiposity. To obtain short-term reliability data, a subset of participants repeated the tasks 3 days later.

Based on previous research with similar figures (e.g., [20]), we hypothesised that participants would be able to successively rank order the bodies in each image set correctly in ascending order from smallest to largest and that they would accurately perceive the differences between successive increments in adiposity, i.e., the stimuli would show validity. Finally, we hypothesised that the responses to the tasks would demonstrate good to excellent test–retest reliability.

## 2. Methods

### 2.1. Sample Size

Our central question asks whether it is possible to detect whether a given body on a scale is heavier or lighter than another. Therefore, to estimate the minimum sample size for the current study, 31 adults completed the visual analogue scale (VAS) detection task used in this study. They viewed images representing the 2nd, 25th, 50th, 75th, 91st, 98th, and 99.6th BMI centiles and indicated the body size of each image using a VAS (extremely underweight to extremely overweight). We calculated the smallest detectable difference in VAS scores between successive centiles (i.e., between the 98th and 99.6th BMI centiles) and used the mean difference score and the standard deviation of these differences to calculate Cohen’s *d*_z_. We then used G*Power version 3.1.9.7, Dusseldorf, Germany [47] to estimate the sample size required to detect this difference with α = 0.01 and power (1 − β = 0.95) using a *t*-test for matched pairs. G*Power returned a sample size of 109 participants; therefore, we recruited until we exceeded this with complete responses.

### 2.2. Participants

We recruited 238 participants who were predominately undergraduate students without children under the age of 18 years. They were recruited using opportunity sampling via social media and [Blanked University’s] SONA system, where they received course credit for their participation. Individuals were eligible to take part if they were over 18, understood written English, and were not currently or previously diagnosed with an eating disorder. A sample of predominantly undergraduates who were not parents was chosen so that participants would be unlikely to have prior extensive experience of interacting with children and thus would be less susceptible to potential expertise effects influencing validation performance. Participants were excluded if they had a current or previous eating disorder diagnosis to avoid any psychological distress from judging bodies. A smaller sub-sample of 151 participants from the original sample completed the follow-up survey three days later to assess test–retest reliability.

### 2.3. Ethics and Pre-Registration

Ethical approval was granted by the Faculty of Health and Life Sciences Research Ethics Committee, Northumbria University (Reference code 120.1867; Approval date: 3 February 2021). This study was pre-registered with the Open Science Framework (OSF): https://osf.io/d7xnu (accessed on 1 October 2024). Data were collected between 22 July 2021 and 16 May 2022.

### 2.4. Stimulus Generation

Jones et al. (2018) [21] utilised 3D surface body scanning technology and captured accurate representations of 388 children aged 4–5 and 10–11 years old to create 3D Body Image Scales (BIS) that have been evaluated as an acceptable visual aid to discuss child weight [21,24]. These age groups were chosen because the English NHS National Child Measurement Programme (NCMP) measures the height and weight of children in schools at these ages as part of its national paediatric weight surveillance programme [48].

All the scan data included in the body shape analyses were from White participants only, as different ethnic groups have different body compositions and patterns of fat deposition (e.g., [44,49,50]). Consequently, they will have different body shapes at a particular BMI. Future studies need to address the issue of creating specific biometrically valid body scales for each of the other ethnic groups as a matter of urgency.

The 3D scans were processed using Wrap3 software (Version 3.3.17, see [51]) and a template base mesh was wrapped around the individual scans by matching 36 pre-selected points (manually located) on corresponding landmarks of both the 3D scan and template model (see [46]). This resulted in all scans having a standardised topology, thereby allowing for statistical comparisons to be made across individual variations in body size and shape. Using customised MATLAB:2024a software, (Massachusetts, USA) the average 3D shape for the set was then calculated, and all individual shapes were subsequently fitted to this average using Procrustes analysis to minimise idiosyncratic differences in body position [46]. Next, each individual shape was entered into a Principal Component Analysis (PCA). The resulting subspace comprised *c* − 1 dimensions, where *c* is the number of scans. For each dimension in the subspace separately, we carried out a linear regression. All participants’ measures of BMI were used to predict their locations along that specific dimension, with the values of the coefficient and the constant subsequently allowing us to model BMI-dependent shape change. Using the results of these regressions, we were able to predict locations along all the subspace dimensions for the BMI values. For the specific location identified in multidimensional space, the 3D shape could then be reconstructed and visualised. Thus, it was possible to create CGI bodies with a specific BMI corresponding to a specific BMI centile point (such as the 50th centile) for a particular age and gender.

To summarise, this analysis isolates the shape change predicted by BMI in this set of bodies and can create the corresponding body shapes at any point along the BMI spectrum. Following previous qualitative work and team discussions, it was deemed that although the 3D physical dimensions of the prototypes were anthropometrically accurate, none looked sufficiently realistic (see [21]). A more realistic representation of the bodies was therefore needed, and so a 3D modelling software package (Daz Studio 4.10 from www.daz3d.com, accessed on 1 October 2024) and the Genesis-8 body models were used to create photorealistic 3D models duplicating the anthropometric dimensions of the MATLAB prototypes (see [40,52]). An advantage of this method is that the same identity of the body in the image for each image set can be maintained over a wide BMI range.

**Figure 1:**
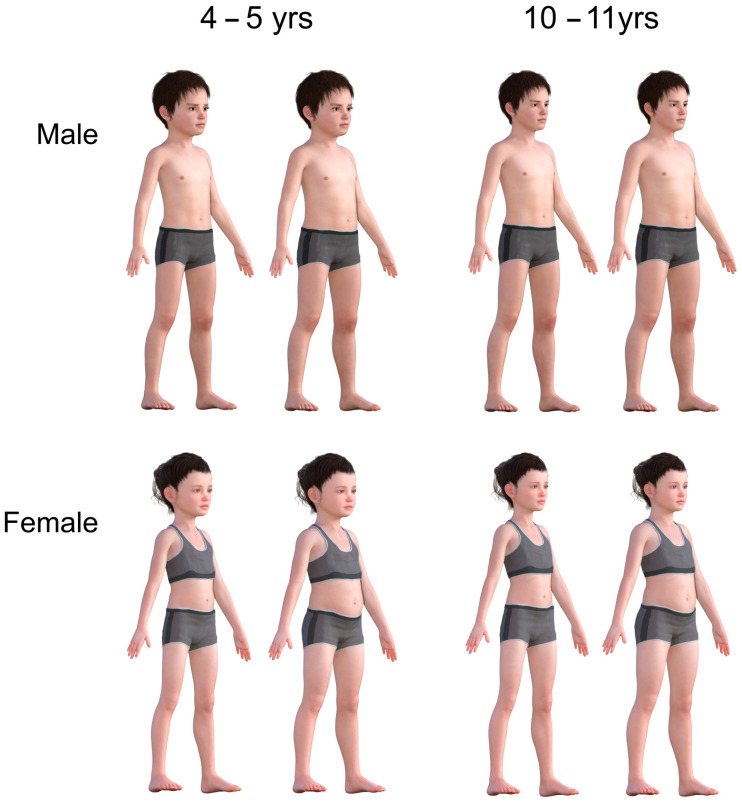
Example stimuli showing younger (4–5 years old) and older (10–11 years old) males and females at the 25th and 75th BMI centiles.

For each set of stimulus images (one set for each gender at each age), seven weight categories were represented: underweight (2nd centile, clinically low weight); lower-healthy weight (25th centile, clinically healthy weight); mid-healthy weight (50th centile, clinically healthy weight); upper-healthy weight (75th centile, clinically healthy weight); overweight (91st centile, clinically overweight), lower-extremely overweight (98th centile, clinically obese) and upper-extremely overweight (99.6th centile, clinically extremely obese). The categories represented were guided by the NCMP’s centile cut-offs and labelling terminology [53]. Examples of the stimuli sets are illustrated in Figure 1.

### 2.5. Procedure

The survey and information/consent documents were hosted online via Qualtrics™. Participants first read an online information form which described the study purpose and methods, right to withdraw, and data storage and handling before then providing informed consent. Before proceeding, participants were asked to confirm that they were using either a laptop or desktop rather than a mobile phone or tablet. If a mobile phone or tablet was being used, the survey was automatically terminated with a request to use either a laptop or desktop computer. This criterion was implemented to avoid respondents viewing the figures on a small screen, which may hinder how well they could perceive the details of the images. If the details were not clearly perceived, the study would be less effective in assessing their capacity to judge their relative weight status.

Participants reported their age, sex (male, female, prefer not to say, prefer to self-describe), weight (in stones and pounds or kilograms) and height (in feet and inches or centimetres). They stated whether they were a student (undergraduate or postgraduate) and, if not, described their current occupation. Next, participants used three separate VAS items to assess the extent to which they believed genetics, overeating, and lack of physical activity caused child obesity (0 = ‘not at all’, 100 = ‘completely’). These items have been used in previous studies to assess participant attitudes to child weight [20].

After this, participants proceeded to complete two stimulus validation tasks for the four child stimulus sets: (1) Rank the images in ascending order from smallest to largest, (2) Rate the body shape/size from extremely underweight to extremely overweight on a VAS (0 to 100). All participants were naïve to the stimuli, having no prior exposure to or knowledge of the images, ensuring unbiased responses when completing the stimulus validation tasks. In the ranking task, all seven bodies from each stimulus set were presented in a random order on the screen at the start of the task. Participants were then asked to move the images into the ascending order of BMI centile. This was achieved using seven numbered boxes (1–7) that participants needed to drag and drop the bodies into. The order of these two tasks was randomised: some participants completed the ranking task first, whilst others completed the VAS task first. Then, within each of the two validation tasks, the order of presentation for the four stimulus sets was also randomised. In the ranking task, all 7 bodies within each stimulus set appeared on the screen at once; however, their order was randomised. In the VAS task, the bodies within each stimulus set were presented individually on the screen, and the order of presentation of each of the 7 bodies within each set was randomised. Therefore, at every level of the study, randomisation was implemented to control for potential biases in image presentation (validation task type, stimulus set and child body BMI centile).

Finally, participants read a debrief sheet with information about the study and related resources. To assess test–retest reliability, all 238 participants were sent the follow-up survey to their email addresses three days later. Of these, 151 repeated the same two body weight perception tasks once more.

## 3. Results

The data files are available through the Open Science Framework (OSF): https://osf.io/gdp9j/ (accessed on 1 October 2024).

### 3.1. Univariate Statistics

We obtained complete datasets for 238 adults (132 women, 105 men, and 1 who preferred to self-describe), all of whom self-reported being White and none of whom had children under the age of 18. The numbers of undergraduate students, postgraduate students, and full-time workers were, respectively, 169, 36, and 33. Participant body mass index (BMI) was calculated (weight/height^2^) after converting self-reported weights to kilograms and heights to metres. Table 1 shows the characteristics of these participants.

### 3.2. Validation of Stimuli: Rank Ordering

We wanted to know whether participants could rank the stimuli in line with their objective size. Table 2 is a confusion matrix showing patterns of match and mismatch between the objective rank order of the stimuli (S), which increased in accordance with the UK90 growth reference [54], and the rank ordering reported by the participant (R). Values in cells show the percentage of stimuli (S1 to S7) in each ranking position distributed amongst the seven possible ranking positions (R1 to R7). Note that there is a 14.3% chance of a correct guess. Shaded cells indicate congruent responses, i.e., the percentage of trials (calculated separately for each row) in which the participants’ chosen rank (R) matched the stimulus’s objective rank (S). Table 2 shows that participant rankings were highly congruent with objective stimulus size. The lowest score in Table 2 was 54.1% correct. A multinomial simulation of a group of 100 participants, guessing the correct rankings across 10,000 resamples, showed that a score of 32% correct for any given weight category was at the 99.99th percentile of the distribution. This strongly suggests that even the worst performance by participants, 54.1%, was significantly better than guessing (i.e., 14% correct) at *p* < 0.0001.

### 3.3. Validation of Stimuli: VAS Ratings

We wanted to know whether participants could detect the directional size differences between stimuli. To achieve this, we analysed how participants assigned VAS values (0 to 100) to stimuli. To minimise the influence of range equalising and centring biases, we normalised the VAS response data [55] per participant and stimulus type. To model these data and test for differences in normalised VAS scores between successive BMI centiles, we used PROC MIXED (SAS v9.4) to build a linear mixed effects model with normalised VAS as the outcome variable. As fixed effects, we tested the following: image (i.e., young girls, young boys, older girls, and older boys), BMI centile (i.e., 2, 25, 50, 75, 91, 98, 99.6), and the interaction between the two. Younger girls and BMI centile 99.6 acted as the control levels for dummy coding. We added random effects at the subject level for intercept, and we used the Satterthwaite method for computing denominator degrees of freedom for the tests of fixed effects. Explanatory variables were retained in the final model if the Type III tests of fixed effects showed that they (a) were statistically significant at *p* < 0.05 and (b) contributed to a statistically significant reduction in −2 log-likelihood. We included participant age and BMI and the three attribution scores (genetic, over-eating and inactivity) as covariants in the analysis.

For the random effect, we found a significant between-participant variance for the intercept (Z = 9.69, *p* < 0.0001). Type III tests of fixed effects showed significant effects for image (F(3, 6398) = 76.40, *p* < 0.0001), BMI centile (F(6, 6398) = 3168.25, *p* < 0.0001), and their two-way interaction (F(18, 6398) = 35.22, *p* < 0.0001). The model parameters are shown in Table S1 of the Supplementary Materials. Table S1 shows that the significant main effect of an image—where younger girls were the controls—was attributable to a significantly lower VAS score for young boys compared to the other three image classes (older boys, younger girls, and older girls). The main effect of BMI centile is due to the systematic increase in VAS scores as a function of BMI centile. (Since BMI centile 99.6 was the control level in the analysis, this is reflected in Table S1 by the increasingly negative difference between the control level and the other 6 BMI centiles). The interaction between image and BMI centile is caused by two factors. First, younger and older boys were assigned systematically higher VAS scores (i.e., ~5 VAS units) than younger and older girls at BMI centiles 2 and 25. But, above BMI centile 50, younger and older girls were assigned systematically higher VAS scores (i.e., ~3–5 VAS units) than younger and older boys. Secondly, on average, younger boys were assigned systematically lower VAS scores (i.e., ~3 VAS units) than older boys, and younger girls were assigned systematically higher VAS scores (i.e., ~1 VAS unit) than older girls. We did not find any statistical effects of any of the five covariants, and they were excluded from the final analysis (and, therefore, do not appear in Table S1). The final model explained 69.8% of the variance in normalised VAS scores relative to the unexplained variance in normalised VAS scores [56,57].

**Figure 2:**
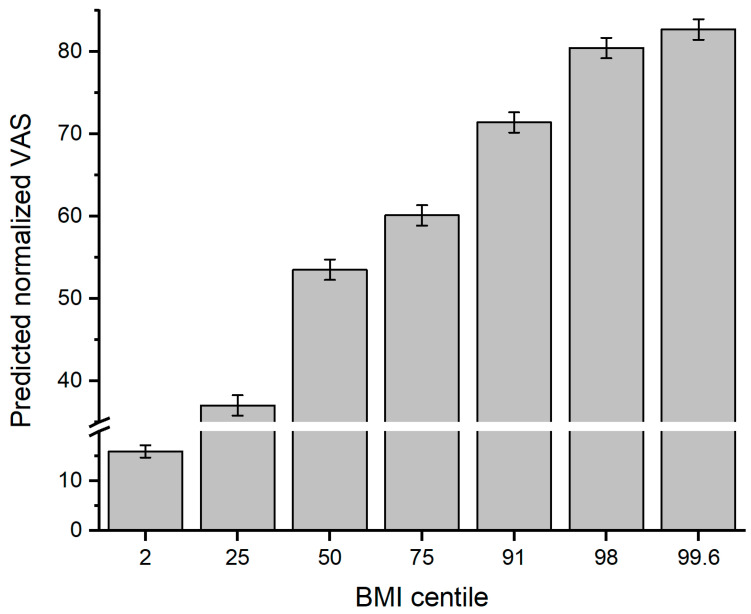
Bar charts of predicted normalised visual analogue figure size score from the model shown in Table S1. Error bars represent 95% CI.

Planned post-hoc comparisons showed that all successive increments in BMI centile, calculated separately for each image type, i.e., BMI centile 2 versus 25, 25 versus 50, 50 versus 75, 75 versus 91, 91 versus 98, and 98 versus 99.6, showed statistically significant differences in normalised VAS scores at *p* < 0.05 or less, suggesting that, on average, participants could successfully discriminate between successive BMI centiles. The predicted mean normalised VAS scores are shown in Figure 2.

### 3.4. Test-Retest Reliability

One hundred and fifty-one participants agreed to a second round of ranking and VAS measurements 3 days after the first round of measurement. For the VAS measurements, we used a mixed-effect, two-way model for absolute agreement to compute intra-class correlations, ICC (2, k), at each BMI centile [58,59]. The results are shown in Table 3 and generally suggest good test–retest reliability.

In addition, we wanted to know how well the sequences of seven VAS measurements agreed over time, relative to each other, rather than whether a given response to one particular stimulus repeated reliably over time (where the ICC would be an appropriate test). For this reason, we also calculated the Pearson correlations between VAS measurements across the seven weight levels at the two time points, separately for GO, GY, BO, and BY image scales and separately for each person. We then calculated the average of these correlations across the sample of 151 participants in each case. The average Pearson correlation between the VAS data for Time 1 and Time 2 was r = 0.86, 95% CI = 0.84–0.97, suggesting good agreement. Finally, the calculated Altmann–Bland plots between the VAS measures at the two time points, separately for each BMI centile, are used to search for bias. The plots are presented in the Supplementary Materials and suggest minimal systematic bias since the mean of the differences (i.e., solid black line) between time points 1 and 2 was always very close to zero.

To examine test–retest reliability for the ranking data, it is inappropriate to calculate intra-class correlations because the seven locations in the rank ordering are not independent of each other, unlike the VAS data. For example, if a participant mislocates one image in the rank, then a second location must also contain an error: e.g., 1, 2, 3, 5, 4, 6, 7. If a participant mislocates two images, then two or three locations will contain an error: e.g., 1, 2, 4, 3, 5, 6, 7 or 1, 2, 4, 5, 3, 6, 7. If a participant mislocates three images, then between three and five locations will contain an error: e.g., 1, 2, 5, 4, 3, 6, 7 or 1, 3, 2, 4, 6, 5, 7, and so on. For this reason, we relied on calculating the Spearman rank correlations across the seven BMI centiles separately for each image type and participant. We then calculated the average of these correlations across the sample of 151 participants. The average Spearman correlation between the ranking data for Time 1 and Time 2 was r_s_ = 0.90, 95% CI = 0.89–0.92, suggesting good agreement.

## 4. Discussion

### 4.1. Summary of Findings

This study validates the use of a new set of photorealistic, anthropometrically accurate body scales for male and female children aged 4–5 and 10–11. Participants can reliably judge and rank the relative size of the bodies on the figural scales, and the test–retest reliability findings suggest that these judgements are consistent and reliable over time. The CGI images of child bodies within these updated scales are based on an analysis of 3D scans of children and so have the anthropometric size and proportions of the real-life bodies of a particular BMI centile that they are meant to represent. Although it has been argued that body scales based directly on 3D scans of real people automatically have face validity derived from this data-driven origin [21,60], this biometric accuracy does not necessarily mean that an observer can actually perceive the difference in size between images within a scale and reliably judge their relative sizes. This study confirms that the relative size of the bodies within this new scale can be discriminated and accurately judged and that the scales can be used as a tool for illustrating weight change and testing body judgements.

Previous studies have used figures presented in front-view or in both front-view and profile [21,43,61,62]. However, a key cue to judging body weight seems to be stomach depth, which is harder to judge in front-view than in profile or three-quarter view [41]. An issue compounded by presenting the stimuli as silhouettes in front-view where all cues to stomach depth are not visible (e.g., [35]). However, simply showing stimuli in profile lacks “ecological validity” (i.e., this is not how someone would usually view and interact with a person whose weight they might judge). Thus, the presentation of body stimuli in three-quarter view in this new scale combines the benefits of a profile presentation with a measure of ecological validity as the face and front of the body are clearly visible (see Figure 1). It is also quicker and easier to administer than the use of two scales with different viewpoints.

The ranking and VAS tasks both suggest it became progressively harder for our participants to discriminate between the bodies as their BMI increased. This could be explained by two simple perceptual biases: contraction bias and Weber’s Law. Body size judgements seem to be made by reference to an internal template based on all the bodies of a particular age and gender that one has seen [63,64]. Such judgements are subject to contraction bias [55]. This means that size judgements are most accurate for bodies of a similar size to the reference but become increasingly inaccurate as the difference between the reference and the body being judged increases. When this happens, the body is judged to be closer in size to the reference than it is. As a result, the size of a body smaller than the reference will be over-estimated, and a body larger will be under-estimated [65]. This compresses the apparent size variation of the bodies within a scale and makes it harder to distinguish between bodies at the extreme ends of the scale. For higher BMI values, this effect is compounded by Weber’s Law. This states that the just noticeable difference (JND) between two bodies will be a constant proportion of their magnitude, leading to a constant Weber fraction over the stimulus range [40,66]. For example, a 10% difference in size between bodies might be required for a distinction to be made. Although a constant proportion (10%) is required to detect a difference between bodies, this will need a bigger absolute difference when the bodies are judged to have a higher BMI as compared to when the bodies have lower BMI. These two perceptual biases will combine to make it harder to discriminate between bodies at the upper end of the BMI spectrum, as is demonstrated in our data.

### 4.2. Future Directions

The figural scales developed and validated in this study have important applications in both research and clinical practice. They can be used to obtain data from families and HCPs on their knowledge, perception, and estimation of children’s body size, as the images in the scale can be directly matched to child BMI values. The scales can be added to epidemiological surveys as a proxy for weight status alongside self-reported height and weight. The scales can also be used to measure a child’s own body image and self-perception. Additionally, they can be used by HCPs to start conversations with parents about their child’s weight status and monitor the progress of weight control interventions [24,31] and as a training tool to improve parents’ perception of child weight status such as in the MapMe intervention which has used these image sets both in paper and online digital form [60]. Existing detection-focused communication tools [25] tend to be based on BMI centile charts, which can be difficult for parents to understand [28]. It also necessitates the measurement of weight and height, which is something that many parents voice concerns about. Image-based resources can be particularly helpful in communicating information rapidly but neutrally, especially when there are language barriers [31]. In addition, both parents and HCPs report primarily relying on visual cues and comparisons to categorise child weight status [32,33] rather than objective measurements of weight and height (e.g., [32,67]). Such body scales have the potential to be a useful tool in weight management interventions [33,60]. Finally, in the UK, they could be used to support the NCMP in the process of feeding back to parents regarding their child’s weight status. Parents are often shocked and in disbelief following receipt of this information and can respond in a very negative way [68].

### 4.3. Study Strengths and Limitations

This study benefited from the recruitment of adults without children, making judgements about stimuli not based on their own child. This acted to minimise emotional/ cognitive factors which might influence how the stimuli are judged and allowed us to explore the basic psychometric properties of the stimuli. This approach ensures that the scale’s psychometric properties are applicable to the general population rather than being skewed by potential expertise effects of those who have prior experience or knowledge of child weight. Repeated discrimination between similar visual stimuli tends to improve a participant’s performance on the task, the so-called expertise effect [69]. Although parents may develop a degree of this expertise due to familiarity with their child’s size and that of the other children in their age cohort, it is unlikely that people within our student-based participant group would have developed this ability. Additionally, requiring participants to make judgements on these stimulus scales, rather than reporting the perceived weight status of their own children, also ensured the standardisation of the procedure. This also provided the opportunity for participants to be tested on multiple weight-related decisions in a way that simply asking parents about their own child’s weight could not.

Previous studies have asked participants to make judgements about body size when presented with figures arranged in ascending size (e.g., [35,43]). This methodology is likely to artificially inflate the test–retest reliability, as participants find it easy to recall which one of the small number of figures in an array that they previously chose [70]. Additionally, body size judgements are influenced by whether they are making judgments about bodies in an ordered versus randomised array [71]. Our study methodology avoids both pitfalls, as in the VAS task, we ask our participants to estimate the size of bodies individually rather than see them in the context of an array of images. Including both the ranking and the VAS task in our study, which was randomised among participants, therefore allowed us to control any effects caused by the way the stimuli are presented on the accuracy of responses.

Although the stimuli that have been validated in this study are a significant improvement on previous scales (e.g., [35,36]), they depict the change in BMI only in White children, resulting in a lack of ethnic diversity. This is because the 3D surface body scan data were collected from predominately White British children living in Northeast England, an area in which almost 96% of inhabitants are White [72]. Previous studies have suggested that differences exist between the body composition (i.e., the proportion of fat to muscle) of people from different ethnic groups [44,45] This has two important implications for the construction of any figural scale. Firstly, the differences in body composition and fat distribution mean that for a given BMI, there will be differences in body size and shape between ethnic groups. Secondly, the cut-offs for weight status are different across the ethnic groups (e.g., [44,45,73]), so the BMI values for the bodies will be different to illustrate these different cut-off values. Thus, although this is a first step in the construction of more accurate realistic figural body scales, further 3D scanning of representative samples of the specific BAME populations and figure creation based on these scans are needed to provide a more ethnically diverse range of figure scales. There are well-documented social and cultural influences on childhood obesity, which may reduce the effectiveness of existing weight management interventions [74,75]. The use of more diverse sets of body scales which are more representative of the target population may improve engagement with minority communities and thus increase the efficacy of weight interventions.

## 5. Conclusions

We have created anthropometrically accurate figural scales based on 3D scans of real children (4–5 and 10–11 years old) with stimuli of known BMI centile values illustrating the UK90 weight status criteria. We have confirmed behaviourally their validity and reliability and suggest that they have great potential for use in both clinical health practice and future childhood obesity-related research.

## Figures and Tables

**Table 1:** Participant characteristics *.

	Women		Men	
	M	SD	M	SD
Age (years)	22.73	7.50	29.39	15.03
BMI (kg/m^2^)	23.70	5.49	25.10	4.08
Genetic attribution	43.15	19.71	31.50	19.70
Overeating attribution	66.12	17.99	67.97	20.37
Inactivity attribution	63.20	22.61	67.91	21.25

* To maintain anonymity, the characteristics of the one participant who preferred to self-describe their gender are not presented separately here.

**Table 2:** Participant responses in the rank ordering task.

	R1	R2	R3	R4	R5	R6	R7
S1	92.1	6.4	0.8	0.3	0.3	0	0
S2	5.7	83.9	7.6	2.1	0.3	0.1	0.3
S3	1.2	6.7	78.4	10.6	2.1	0.6	0.4
S4	0.3	2	10.4	74.3	9.6	1.7	1.8
S5	0.3	0.6	1.6	9.7	64.4	15.2	8.1
S6	0.1	0.4	0.8	2.4	14.9	54.1	27.2
S7	0.1	0.1	0.4	0.4	8.4	28.3	62.3

Note. S = stimulus; R = response; S1 = 2nd centile, S2 = 25th centile, S3 = 50th centile, S4 = 75th centile, S5 = 91st centile, S6 = 98th centile, S7 = 99.6th centile, R1–R7 = ranking positions. The grey highlights indicate the position of the correct response on each trial (i.e., S1 should be ranked as R1, and so on).

**Table 3:** Test–retest intra-class correlations.

BMIc	ICC	95% CI	Interpretation
2	0.88	0.85–0.91	Good
25	0.74	0.67–0.81	Good
50	0.66	0.56–0.74	Moderate
75	0.63	0.52–0.71	Moderate
91	0.75	0.66–0.80	Good
98	0.86	0.82–0.90	Good
99.6	0.83	0.78–0.87	Good

## Data Availability

This study was pre-registered with the Open Science Framework (OSF): https://osf.io/d7xnu (accessed on 1 October 2024). Data were collected between 22 July 2021 and 16 May 2022.

## Supplementary Materials

The following supporting information can be downloaded at: https://www.mdpi.com/article/10.3390/children11101243/s1, Supplementary Materials containing details of the VAS data analysis (S1) including the Altmann-Bland plots.
