# Supplementary material for: The MapMe Body Scales: Validity and Reliability of a Biometrically Accurate, Photorealistic Set of Child Body Size Scales
Source: Children (Basel). 2024 Oct 16;11(10):1243. doi: 10.3390/children11101243 (PMC11506425; doi:10.3390/children11101243)
Supplement: Supplementary file 1 [file children-11-01243-s001.zip › children-3158040-supplementary.pdf]

Table S1: Linear Mixed Effects model parameters from normalized VAS data

×

Effect	Image	BMIc	Estimate	SE	t Value	DF	p value	95% CI	
Intercept			85.34	0.98	87.31	2800	<.0001	83.42	87.25
Image	BO		-1.74	1.22	-1.43	6398	0.2	-4.13	0.64
Image	BY		-7.96	1.22	-6.54	6398	<.0001	-10.34	-5.57
Image	GO		-0.88	1.22	-0.72	6398	0.5	-3.27	1.50
Image	GY		0	.	.	.	.	.	.
BMIc		2	-71.049	1.22	-58.40	6398	<.0001	-73.43	-68.66
BMIc		25	-51.51	1.22	-42.33	6398	<.0001	-53.89	-49.12
BMIc		50	-27.42	1.22	-22.53	6398	<.0001	-29.80	-25.031
BMIc		75	-19.00	1.22	-15.62	6398	<.0001	-21.38	-16.61
BMIc		91	-12.27	1.22	-10.080	6398	<.0001	-14.65	-9.88
BMIc		98	-0.52	1.22	-0.42	6398	0.7	-2.90	1.87
BMIc		99.6	0	.	.	.	.	.	.
Image × BMIc	BO	2	12.20	1.72	7.090	6398	<.0001	8.83	15.57
Image × BMIc	BO	25	13.69	1.72	7.96	6398	<.0001	10.32	17.064
Image × BMIc	BO	50	-2.97	1.72	-1.72	6398	0.1	-6.34	0.41
Image × BMIc	BO	75	-7.63	1.72	-4.44	6398	<.0001	-11.008	-4.26
Image × BMIc	BO	91	-3.19	1.72	-1.85	6398	0.06	-6.56	0.19
Image × BMIc	BO	98	-2.42	1.72	-1.41	6398	0.2	-5.79	0.95
Image × BMIc	BO	99.6	0	.	.	.	.	.	.
Image × BMIc	BY	2	8.83	1.72	5.13	6398	<.0001	5.46	12.20
Image × BMIc	BY	25	12.57	1.72	7.31	6398	<.0001	9.20	15.94
Image × BMIc	BY	50	-1.65	1.72	-0.96	6398	0.3	-5.027	1.72
Image × BMIc	BY	75	-3.76	1.72	-2.18	6398	0.03	-7.13	-0.39
Image × BMIc	BY	91	-1.40	1.72	-0.81	6398	0.4	-4.77	1.97
Image × BMIc	BY	98	-2.14	1.72	-1.24	6398	0.2	-5.51	1.23
Image × BMIc	BY	99.6	0	.	.	.	.	.	.
Image × BMIc	GO	2	-3.70	1.72	-2.15	6398	0.03	-7.079	-0.33
Image × BMIc	GO	25	-2.79	1.72	-1.62	6398	0.1	-6.17	0.58
Image × BMIc	GO	50	-2.41	1.72	-1.40	6398	0.2	-5.78	0.96
Image × BMIc	GO	75	-2.90	1.72	-1.68	6398	0.09	-6.27	0.48
Image × BMIc	GO	91	8.48	1.72	4.93	6398	<.0001	5.10	11.85
Image × BMIc	GO	98	-2.37	1.72	-1.38	6398	0.2	-5.74	1.0043
Image × BMIc	GO	99.6	0	.	.	.	.	.	.
Image × BMIc	GY	2	0	.	.	.	.	.	.
Image × BMIc	GY	25	0	.	.	.	.	.	.
Image × BMIc	GY	50	0	.	.	.	.	.	.
Image × BMIc	GY	75	0	.	.	.	.	.	.
Image × BMIc	GY	91	0	.	.	.	.	.	.
Image × BMIc	GY	98	0	.	.	.	.	.	.
Image × BMIc	GY	99.6	0	.	.	.	.	.	.

Altman Bland plots for test retest VAS data

Altman-Bland plots for each BMI centile are shown below. In each case the y-axis represents the difference in VAS scores between time points 1 and 2 (T1 and T2), while the x-axis represents the mean of the VAS scores at times points one and two. The solid black line represents the overall mean difference score between T1 and T2. The upper dashed line is this mean + 2 SD. The lower dashed line is the mean - 2 SD.

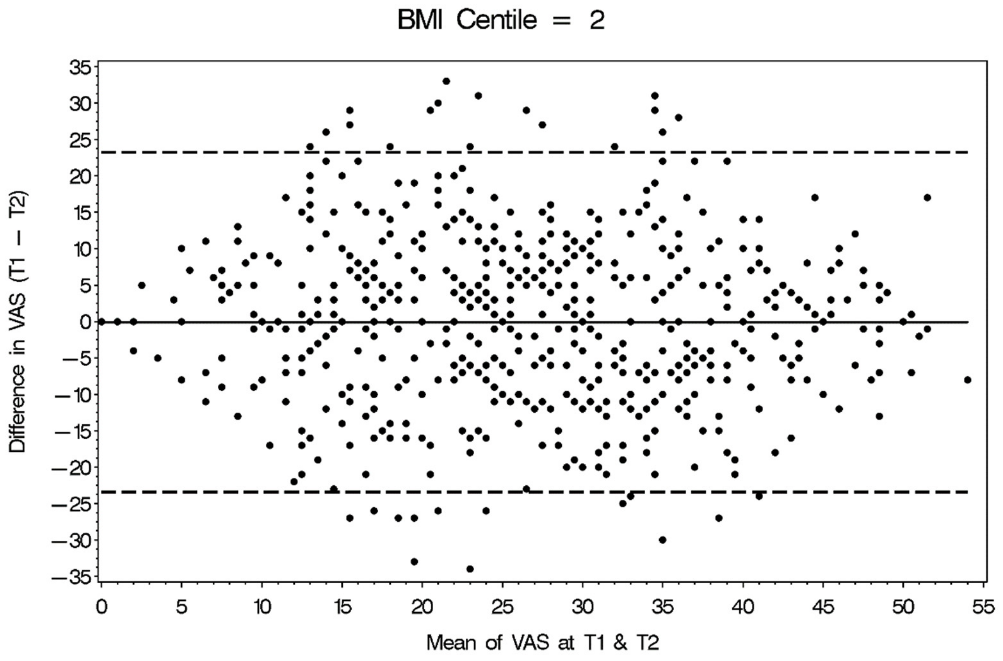

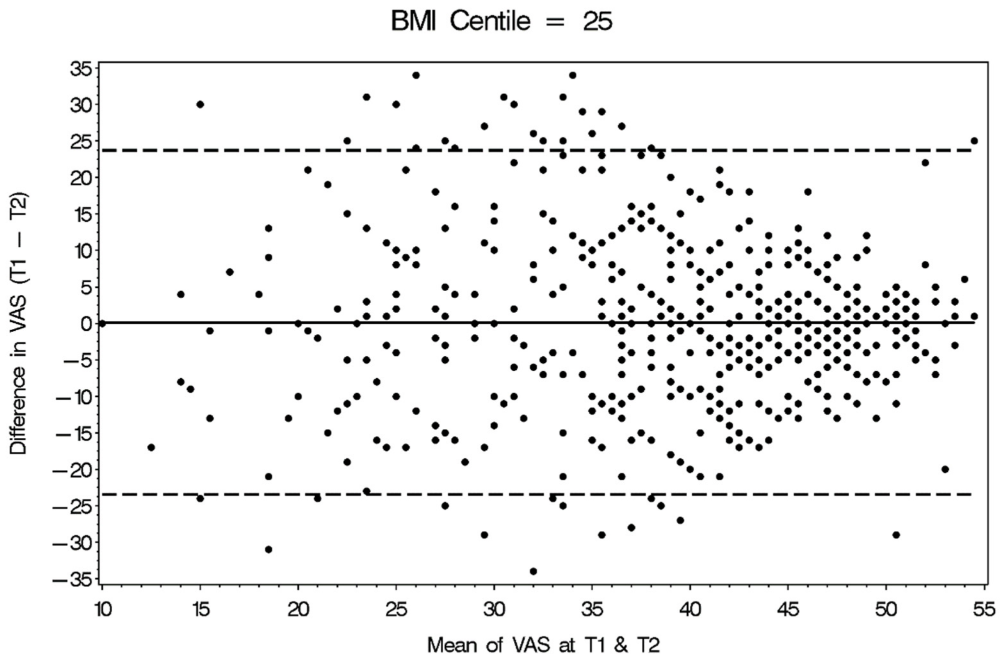

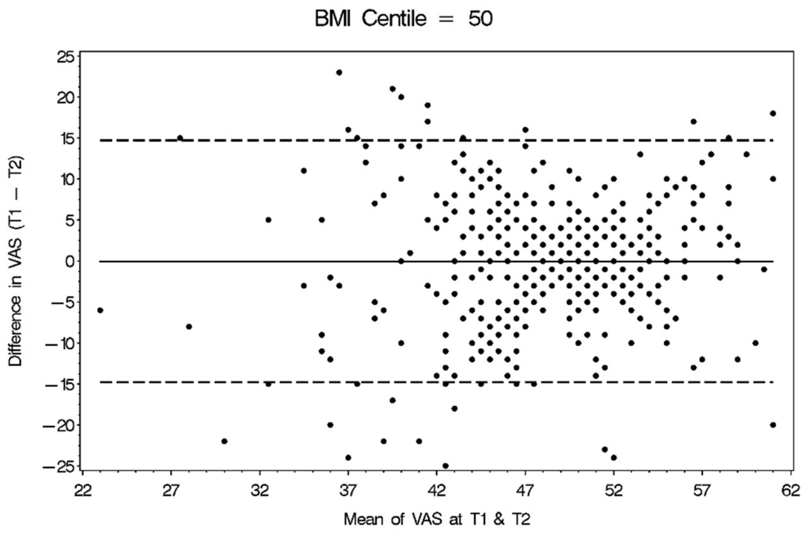
BMI Centile = 50
Difference in VAS (T1 − T2)
Mean of VAS at T1 & T2

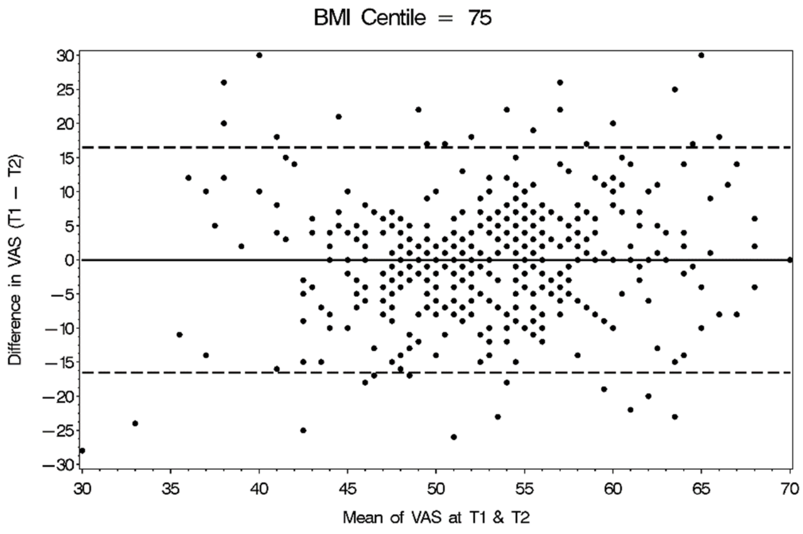
BMI Centile = 75
Difference in VAS (T1 − T2)
Mean of VAS at T1 & T2

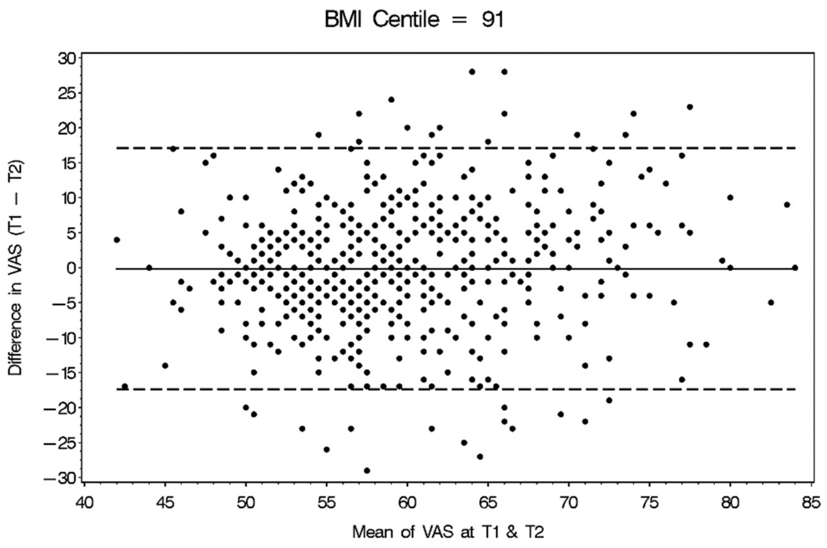
BMI Centile = 91
Difference in VAS (T1 − T2)
Mean of VAS at T1 & T2

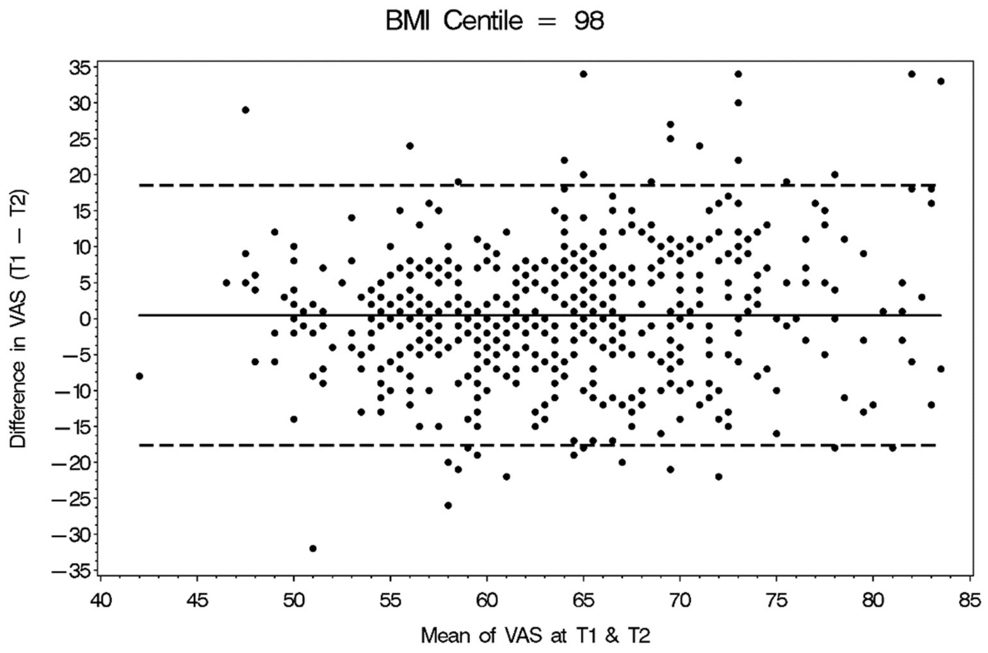
BMI Centile = 98
Difference in VAS (T1 − T2)
Mean of VAS at T1 & T2
35
30
25
20
15
10
5
0
−5
−10
−15
−20
−25
−30
−35
40
45
50
55
60
65
70
75
80
85

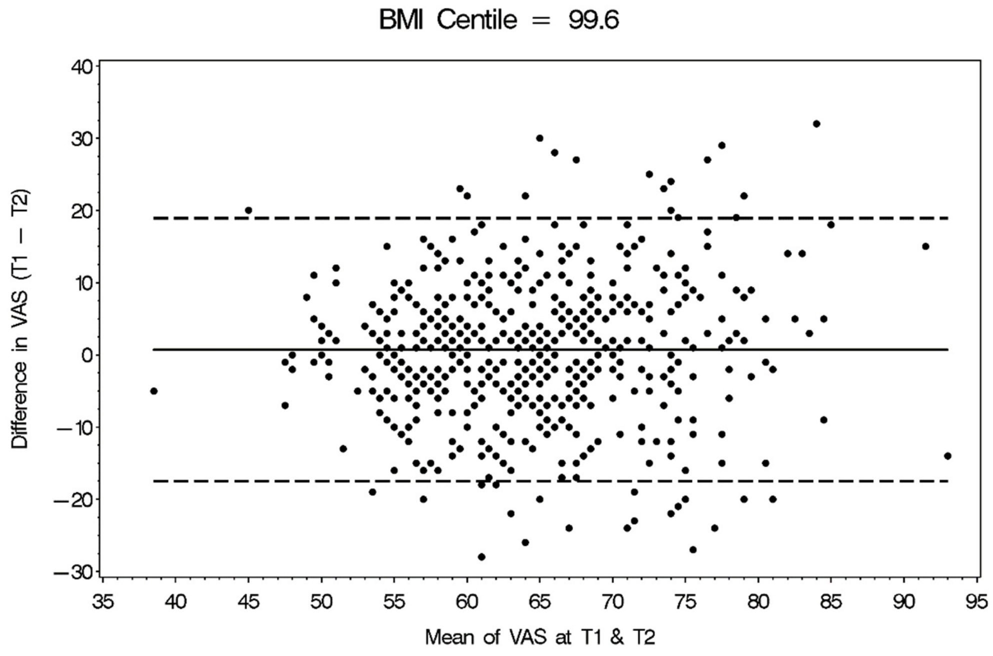
BMI Centile = 99.6
Difference in VAS (T1 − T2)
Mean of VAS at T1 & T2
40
30
20
10
0
−10
−20
−30
35
40
45
50
55
60
65
70
75
80
85
90
95
